# Adoption of potato varieties and their role for climate change adaptation in India

**DOI:** 10.1016/j.crm.2019.01.001

**Published:** 2019

**Authors:** Willy Pradel, Marcel Gatto, Guy Hareau, S.K. Pandey, Vinay Bhardway

**Affiliations:** a Social and Nutrition Science Division, International Potato Center, 15024 Lima 12, Peru; b Social and Nutrition Science Division, International Potato Center, Pham Van Dong Street, Tu Liem, Hanoi, Viet Nam; c ICAR-Central Potato Research Institute, New Delhi, India; d Division of Crop Improvement, ICAR-Central Potato Research Institute, Bemloe, Shimla 171001, Himachal Pradesh, India

**Keywords:** Climate change, Resilience, Potato varieties, India, Adoption study

## Abstract

Adoption of improved varieties is an important strategy to adapt to the negative implication associated with climate change and variability. However, incomplete data on varietal release and adoption is often the reality in many countries hindering informed decision-making on breeding and varietal dissemination strategies to effectively adapt to climate change. In taking the example of potatoes in India, we analyze the extent to which the potato sector is resilient to climate change. We do so by comparing state-level climate change projections with adoption of high resistant and tolerant potato varieties to major abiotic and biotic stresses. Release and adoption data was collected in 2016 in six expert elicitation workshops conducted with 130 experts from the potato value chain in Bihar, Gujarat, Karnataka, Punjab, Uttar Pradesh, and West Bengal. We found that from the total of 81 releases, 45 improved varieties are adopted in India and that in each state high resistant and tolerant varieties are cultivated providing some degree of varietal resilience. Early maturity has been the most important and heat tolerance is the least important trait. Comparing climate projections with adoption rates of high resistant and tolerant varieties, we found that Gujarat is relatively most resilient. In other states we found some mismatches between climate projections and adopted specific varietal traits. Our results allow policy-makers and breeders to better prioritize investments into breeding for specific traits and dissemination strategies.

## 1 Introduction

Climate change has profound effects on agricultural productivity on a global scale (Kang et al., 2009). India is expected to be adversely affected by climate change and variability (Kumar et al., 2015). Temperatures are projected to rise by 0.5 °C by 2030, resulting in fewer rainy days and more extreme weather events, such as prolonged droughts (NIC, 2009). If left unaddressed, climate change and variability may undermine rural incomes and food security in India by longer spells of water shortages and increased incidence of pest and diseases (Singh et al., 2013, Sparks et al., 2014, Peet and Wolfe, 2000, Mall et al., 2006).

Potatoes, in addition to cereals, contribute largely to food security in India (Singh and Rana, 2013, Naitam et al., 2017). In 2016, potatoes in India occupied an area of 2.13 million hectares, total annual production reached almost 44 million tonnes and yields averaged 20.5 tonnes per hectare (Directorate of Economics and Statistics, 2017). With a projected population increase of 19% by 2050 (United Nations, 2017), India faces a tremendous challenge to increase production of all food crops, including potatoes, to meet future demands.

Without adaptation to climate change and other mitigation to technological adaptation gaps, simulations project a 23% decline in potato yields by the years 2040–2059 (Hijmans, 2003). Other simulations are less severe, projecting a yield reduction of ‘only’ 2.5–13.72% between 2020 and 2050 (Singh and Lal, 2009, Kumar et al., 2015). Due to India’s size and agro-ecological diversity, climate change and variability affect India’s states differently. For example, seasonal temperature increases beyond the optimum have negative effects on yields particularly in Central and Eastern India (Dua et al., 2013, Haris et al., 2015). On the other hand, in the north-western parts of the country, yield gains are expected as temperatures move towards optimal levels from current low temperatures (Singh and Lal, 2009). The temperate Indian hills are highly susceptible to severe epidemics of late blight, but the disease now appears earlier in the northern part (November) and later in the eastern part (February) and within a wider temperature range (Gautam et al., 2013).

Adaptation to climate change is crucial for increasing yields or, at best, maintaining yields at current levels. Various strategies exist, such as shifting cultivation to cooler seasons, increasing fertilizer application to compensate for increased loss at higher temperatures (Kumar et al., 2015), mulching, or implementing water and soil management strategies and pest and disease management strategies (Sparks et al., 2014, Singh and Lal, 2009, Thomas-Sharma et al., 2016). The adoption of improved crop varieties is another important adaptation strategy. Using early-maturing varieties, which mature between 70 and 90 days, allows for more flexibility in planting or harvesting the crop and potentially provides additional income if cultivated in between two rice cycles (Bardhan Roy, et al., 2007). Other relevant varietal traits are resistances and tolerances to biotic and abiotic stresses, such as heat and drought tolerance (Islam et al., 2016). Take the example of heat stress, which poses a major threat to potato production, due to the delay in tuber initiation, malformation and necrosis of tubers (Levy and Veilleux, 2007). In addition, drought may affect potato production, not only by limiting the plant to absorb water, but also by increasing the salt concentration in the soil, which affects the reverse osmosis of water loss from plant cells (Basu et al., 2016). Furthermore, late blight and virus resistance will become increasingly important traits as diseases evolve (Chowdappa et al., 2015) and pests and pathogens spread more freely (Bebber et al., 2014).

The objective of this paper is to understand the extent that the potato sector in India has adapted to climate change by focusing on improved varieties as an adaptation strategy. We first analyze the current stock of released and adopted improved varieties with a focus on abiotic tolerance levels, late blight resistance, and maturity. Second, in acknowledging that the adverse effects of climate change differ by region, we examine how potato production is affected at the state level, focusing on six important potato producing states (i.e. Bihar, Gujarat, Karnataka, Punjab, Uttar Pradesh, West Bengal). In doing so, we developed and applied a Varietal Resilience Indicator based on adoption estimates and varietal trait information.

Our research intends to inform policy-makers and national breeding programs by, first, taking stock of state-level adoption rates of potato varieties with high resistance and tolerance levels, which are not well-documented but critical in adapting to climate change. Second, our results will support policy-makers to prioritize the dissemination of specific varieties at the state level in our study region. Lastly, this study provides trait-level insights and adoption estimates that may be useful in shaping future breeding agendas.

## 2 Background

India experienced one of the major shifts towards food sovereignty to include potato as an important part of food security policy as a rice diversification effort (Walker et al., 1999). After India’s independence in 1947, India supported the creation of an ICAR-Central Potato Research Institute (CPRI) to develop locally adapted varieties. The first set of varieties was released in 1958. Thereafter in the sixties, India established a potato seed system called “Seed Plot Technique” to increase potato seed production in the country (Singh, 2014). Astonishingly, the policy helped to increase production from 1.5 million tonnes in 1950 to more than 46 million tonnes in 2014 (Singh, 2014, Sharma, 2014). The phenomenal increase in productivity and production of potatoes has been termed as the “Brown Revolution” that placed India as the second major potato producer in the world (Bamberg and del Rio, 2005).

Potatoes are grown across India under diverse agro-climatic conditions in almost all states except the coastal belt (Table 1). Nearly 82% of potatoes are grown in northern India, in the vast Indo-Gangetic Plain (IGP) during short winter days from October to February/March. In this context, the duration of the thermally suitable window is the main determinant limiting yields for potatoes (Kumar et al, 2015).

**Table 1:** Potato: area, production and yield in major producing states, 2015–2016 cropping season.

State	Area ('000 has)	Production ('000 t)	Yield (t/ha)
Uttar Pradesh	607.32	13,851.76	22.81
West Bengal	427.00	8,427.00	19.74
Bihar	319.13	6,345.52	19.88
Madhya Pradesh	141.05	3,161.00	22.41
Gujarat	112.40	3,549.38	31.58
Assam	104.83	1,037.26	9.89
Punjab	92.99	2,389.48	25.70
Karnataka	48.08	651.45	13.55
Other States	280.77	4,356.72	15.52

India (total)	2,133.58	43,769.56	20.51

*Source:*Directorate of Economics and Statistics (2017).

The duration of a thermally suitable window available for potato cultivation varies greatly within the IGP region. States in the north-west like Punjab and the Western Uttar Pradesh are characterized by relatively harsh long winters in the sub-tropical plains of India. Punjab is known as the ‘Seed Bowl’ of the country because of absence or low presence of aphids during October. Since the Central IGP has milder winters, agriculture intensification is high, and farmers frequently cultivate three crops in a year. The Eastern IGP has two major states: West Bengal and Bihar. West Bengal is the second largest potato producing state in India after Uttar Pradesh, and water is in abundance where the crop is grown in the alluvial deltaic soil of the longest river, the Ganges. In contrast, Bihar is one of the poorest states and home to many smallholder potato farmers, owning on average less than 1 acre. Low potato productivity in this region is mainly due to highly degenerated varieties, lack of mechanization, and poor storage facilities. Water deficiency is an issue in western States such as Gujarat and Karnataka. However, Gujarat utilizes drip irrigation for cultivation while Karnataka relies on rain during the rainy season from June to September. Additionally, Karnataka potato producers suffer significant losses due to late blight (Rana et al., 2013a).

## 3 Materials and methods

### 3.1 Data

For this study, we draw on two databases, one on varietal release and one on varietal adoption. The release database was established through CPRI and includes details on variety names, year of release, pedigree, institutional source, maturity levels, and various pest-resistance and disease-tolerance levels. This database is publicly available (Gatto et al., 2016a) and was established in 2015; any releases which occurred after that were not captured and thus are not considered in this study. Due to India’s centralized varietal release system, it was not necessary to collect varietal release data for each individual state. The varietal release data provided by CPRI was cross-checked at the beginning of the expert elicitation workshops, which are explained in the following paragraphs.

The varietal adoption database was established using expert elicitation workshops following a well-established, cost-effective methodology, which has been widely applied (e.g. for rice in South Asia see Tsusaka et al. (2015); for Sub-Saharan Africa see Walker and Alwang (2015); for wheat in India see Pavithra et al. (2017); for potato in Asia see Gatto et al. (2018); for maize in India see Pavithra et al. (2018). In more detail, adoption estimates were elicited following a standardized 5-step procedure, which are described in detail in Gatto et al. (2018).

Expert elicitation workshops were organized in each of the sampled six major potato producing states in India: Bihar, Gujarat, Karnataka, Punjab, Uttar Pradesh, and West Bengal. Our study region is representative of 75% of the India’s total potato cultivation area (Table 1).

In 2016, we organized the six workshops with a total of 130 experts participating, which averages 21.7 experts per workshop. To ensure a high degree of representativeness, participants were experts working in the potato sector including farmers and seed producers, government officials, breeders, extension agents, cold storage owners, representatives from processing and seed industries, and NGO representatives and traders. Varietal adoption estimates refer to the year 2015.

### 3.2 Varietal resilience indicator

To analyze the extent to which Indian states are adapting to climate change by focusing on improved varieties, we developed a Varietal Resilience Indicator. This indicator uses the release data for crop maturity, heat and drought tolerance, and late blight resistance at the varietal level. The Drought Tolerance Index (DTI) is based on the Modified Stress Tolerance Index (Kumar and Minhas, 2013), while the Heat Tolerance Index (HTI), Maturity Index (MI), and Late Blight Resistance Index (LBI) are based on potato properties from varieties produced by CPRI (Kumar et al., 2014) and corroborated with expert knowledge from the expert elicitation workshops. More formally, the Varietal Resilience Indicator is calculated as follows:

(1) $$\mathrm{Varietal}\;Resilience_{\mathrm{ts}}=\;\sum \left(\frac{\mathrm{Are}a_{\mathrm{is}}}{\mathrm{TotAre}a_{s}}\right)\times \;Level_{\mathrm{tis}}$$

where $\mathrm{Varietal}\;Resilience_{\mathrm{ts}}$ is the varietal resilience indicator of trait t (i.e. maturity, heat, drought, and late blight) in State s in 2015; $\mathrm{Are}a_{\;}$ is the area in hectares cultivated by variety i in State s in 2015; TotArea is the total area in hectares cultivated by potatoes in State s in 2015; Level of trait t of variety i in State s in 2015 refers to the categories assigned to measure trait variances. For the varietal trait maturity, the categories are 0 (late), 1 (medium late), 2 (medium), and 3 (early). For the remaining traits – heat, drought, and late blight - the categories are 0 (sensitive), 1 (low), 2 (medium), and 3 (high).

For easier interpretation, we normalized the Varietal Resilience Indicator to range between 0 and 1. Values closer to zero may be interpreted as lower adoption rates of high resistant and tolerant, and early-maturing varieties on a state-wide level; sensitive or late-maturing varieties are likely to dominate. In these cases, climatic resilience provided by varieties is low. In contrast, values closer to 1 represent higher varietal resilience on a state-wide level.

## 4 Results

### 4.1 Release of potato varieties in India

Up until 2015, this study identified a total of 81 varieties released or introduced in India’s national release system, where 50 varieties come from CPRI, 22 varieties from developed countries’ agricultural research agencies (mainly from United Kingdom, Netherlands and United States), and another 9 varieties were either local varieties or of unknown origin (Gatto et al., 2018).^1^ Generally, these varieties are widely adapted to India’s different agro-climatic conditions, from the very harsh long winters in sub-tropical plains to the southern peninsular region characterized by dry and warm weather.

According to our release database, the first varieties developed by CPRI were released in 1958, and after that breeding efforts have increased and resulted in the release of 50 varieties. Most of the varieties released in India are medium-maturing, sensitive to heat and have a medium resistance to late blight. Most of the varieties are equipped with one specific trait, while a select few have multiple traits (i.e. high resistance/tolerance, early-maturing), as Table 2 depicts.

**Table 2:** List of varieties released in India indicating their main climate change related attributes and preferences to Indian farmers.

Variety name	Yield potential (t/ha)	Crop maturity^1^	Heat tolerance	Drought tolerance	Late blight resistance
*Most popular varieties*					
KUFRI PUKHRAJ	35–40	EARLY	SENSITIVE	HIGH	MEDIUM
KUFRI JYOTI	25–30	MEDIUM	SENSITIVE	MEDIUM	MEDIUM
KUFRI BAHAR	30–35	MEDIUM	SENSITIVE	MEDIUM	SENSITIVE
BHURA ALOO	24	LATE	LOW	SENSITIVE	SENSITIVE
KUFRI CHIPSONA	30–35	MEDIUM	SENSITIVE	MEDIUM	HIGH
KUFRI SINDHURI	30–35	LATE	HIGH	MEDIUM	SENSITIVE
KUFRI CHANDRAMUKHI	20–25	EARLY	SENSITIVE	HIGH	SENSITIVE
KUFRI KHYATI	25–30	EARLY	SENSITIVE	HIGH	HIGH

*Most promising varieties*					
LADY ROSETTA	30	EARLY	HIGH	HIGH	SENSITIVE
KUFRI MEGHA	25–30	MEDIUM	SENSITIVE	MEDIUM	HIGH
KUFRI KANCHAN	25–30	MEDIUM	SENSITIVE	MEDIUM	MEDIUM
KUFRI ARUN	30–35	MEDIUM	SENSITIVE	HIGH	HIGH
KUFRI SURYA	25–30	EARLY	HIGH	MEDIUM	SENSITIVE

Notes: ^1^Early: 70–90 days, Medium: 90–100 days, Medium late: 100–110 days, Late: > 110 days; a selection of further reading on varietal traits and varieties include Kumar et al. (2014) on yield potential, late blight resistance and crop maturity; Kumar and Minhas, 2013, Sharma et al., 2014 on drought tolerance and late blight resistance; Kumar and Sinha (2009) on Bhura Aloo; Sadawarti et al., (2018) on Kufri Khyati; Patel et al. (2005) and Minhas et al. (2006) on Kufri Surya.

*Source:*Gatto et al., 2016a, Gatto et al., 2018.

### 4.2 Varietal adoption and adaptation to climate change

Despite the high number of releases, only 45 varieties were found to be cultivated in our study area in 2015. In addition to national releases, foreign varieties have been introduced to the country and adopted. The most prominent varieties are Lady Rosetta, Atlantic, and Desiree which are being grown in the states of West Bengal and Gujarat. Foreign released varieties like Cardinal, Diamant, Kennebec, Innovator, Santana, Shepody, etc., which are only grown in small pockets in Uttar Pradesh, Gujarat, and West Bengal, are used mostly for processing. Despite its known late blight susceptibility, the industry pushes the use of certain foreign varieties such as Atlantic (Rana et al, 2013b).

Different sets of varieties are adopted in our sampled states according to needs and seed availability. In what follows, we provide information on some of the most dominating varieties, based on area under cultivation. Table 3 summarizes the information and presents the most dominating varieties by state.

**Table 3:** Most adopted potato varieties in six Indian states in 2015.

	Variety	Area (has)	Percentage of total area^1^
*India (6 States)*	Kufri Pukhraj	521,375	33%
	Kufri Jyoti	325,665	21%
	Kufri Bahar	272,642	17%

*Bihar*	Kufri Pukhraj	121,464	39%
	Bhura Aloo	70,539	22%
	Kufri Sindhuri	31,082	10%

*Gujarat*	Kufri Pukhraj	95,630	85%
	Kufri Khyati	10,706	10%
	Kennebec	2192	2%

*Karnataka*	Kufri Jyoti	38,993	94%
	FL-1533	1170	3%

*Punjab*	Kufri Pukhraj	57,411	64%
	Kufri Jyoti	14,625	16%
	Lady Rosetta	3600	4%

*Uttar Pradesh*	Kufri Bahar	271,328	45%
	Kufri Pukhraj	167,176	28%
	Kufri Chipsona 1	59,526	10%

*West Bengal*	Kufri Jyoti	228,539	56%
	Kufri Pukhraj	79,321	19%
	Kufri Chandramukhi	30,919	8%

Note: ^1^for 6 states which represents 75% of total potato area in India.

*Source:*Gattoo et al., 2016b, Gatto et al., 2018.

Kufri Pukhraj (released in 1998) is a high yielding, early-maturing variety and is, by far, India’s most dominating variety covering 521,375 ha (or 33% of total potato area) in 2015. In Punjab, Gujarat, and Bihar it is the most dominating variety, while in Uttar Pradesh and West Bengal it is the second most dominating variety. Only in Karnataka is it of no considerable importance. This variety benefits producers because it is early-maturing and produces sizeable yields even if harvested pre-maturely after 60 days; however, it showed limited yield potential under early sowing condition and is sensitive to heat (Kumar et al., 2014).

The second most important variety cultivated in India is Kufri Jyoti (released in 1968) covering 325,665 ha (or 21% of total potato area) in 2015. It is the dominant variety in Karnataka and West Bengal in 2015, and second most important variety in Punjab. Despite erosion in its resistance to late blight, susceptibility to cracking, and lower yields compared to, for instance, Kufri Pukhraj, it is still preferred in several states because of good shape and storability, size of tubers, and a slow degeneration rate (Kumar et al., 2014).

The third most important variety is Kufri Bahar (released in 1980) which covers 272,642 ha (or 17% of total potato area). It is most popular in Uttar Pradesh; however, it is susceptible to late blight and yields are moderate.

Another important variety, particularly in Bihar, is Bhura Aloo, which is a native variety cultivated with low productivity and late blight susceptibility. Farmers’ preferences for red skinned potatoes keep the demand high for Bhura Aloo, as well as Kufri Sindhuri and Lal Gulal.

Notable varieties used for industrial purposes are Kufri Chipsona 1, Kufri Chipsona 3, and foreign varieties such as Atlantic, Kennebec and Lady Rosetta. Kufri Chipsona 1 (released in 1998) is primarily found in Uttar Pradesh and is suitable for the industry due to its low sugar content, high dry matter, and good tuber size and shape. However, it suffers from heat susceptibility, is moderately susceptible to drought, but highly resistant to late blight.

### 4.3 Effects of climate change on potato production in India

Potato production is most likely affected by climate-related changes in temperature, rainfall patterns, and indirect effects, such as higher severity and incidence of pest and disease outbreaks. Even though predictions of climate change in Indian agriculture are still uncertain, some authors anticipate that increases in weather extremes will severely impact India (Mall et al., 2006). However, due to India’s size, regional differences exist on how climate change affects potato production. Table 4 shows an overview of the expected effects which are heterogenous and state-dependent.

**Table 4:** Expected effects of climate change on temperature, rainfall, and potato yield with no adaptive measures by state.

State	Temperature	Rainfall	Effect on potato yield
*Bihar*	Increasing trend	Mixed trend	Negative
*Gujarat*	Increasing trend	Increasing trends	Negative
*Karnataka*	Increasing trend	Mixed trends	Negative
*Punjab*	Decreasing trend	Decreasing trend	Mixed
*Uttar Pradesh*	No change	Mixed trend^1^	Mixed
*West Bengal*	Increasing trend	Mixed trend	Negative

Notes: ^1^Positive in western parts of Uttar Pradesh, negative in eastern parts of Uttar Pradesh; a detailed table showing the specific effects and their predicted magnitudes and associated literature can be found in Appendix 1.

In northern states like Punjab and Western Uttar Pradesh, which currently have the lowest average minimum temperatures, are not expecting increase in temperature, and some authors expect increase in rainfall as well; as a result, potato production may benefit in those regions. Nonetheless, in Punjab, in addition to the monsoon, farmers also utilize groundwater sources for irrigation and there is evidence of overexploiting this resource (Baweja et al., 2017), and further depletion of groundwater may reduce the benefits of the increase rainfall. Southern states like Gujarat and Karnataka, where temperatures are already high, may be severely affected due to increased temperatures and late blight incidence (Singh and Lal, 2009). Punjab is expected to be less warm but rainier, which is beneficial for potato production, but late blight and other pests and diseases will also become more problematic. A similar trend will likely occur in Western Uttar Pradesh. In other parts of the country, the increasing temperatures will potentially jeopardize potato production. This will especially be the case in Karnataka where potato production will become a risky endeavor.

Currently in Bihar, minimum temperatures are rising significantly (Haris et al., 2015) approaching the upper threshold limits for many crops and planted varieties (Tesfaye et al., 2017). In Punjab, increases in atmospheric CO_2_ concentration levels may compensate for increases in temperatures, resulting in overall stable potato yields (Dua et al., 2013).

Another problem related to climate change is the reduction in rainfall. Regions highly dependent on monsoons, such as IGP, will be affected. The states most affected by enhanced stress of lower moisture due to reduced rainfall trends will be Bihar and eastern Uttar Pradesh, Gujarat, and Karnataka, while West Bengal and western Uttar Pradesh are expected to be positively impacted by rain coming from monsoons (Guhathakurta and Rajeevan, 2008, Kumar et al., 2012, Mall et al., 2006, Tesfaye et al., 2017). O’Brien et al., (2004) identified that states with higher dependence on monsoons and higher drought sensitivity are those in the north-western peninsula (which includes Punjab and Gujarat). However, Guhathakurta and Rajeevan (2008) predict an increase in rainfall for those states, and Mohanty et al. (2015) found an increase in rainfall extremes in Gujarat. The relatively wealthier states in our study region face fewer constraints such as better access to markets, better rural electrification and lower percentage of marginal farmers, therefore being better equipped to adapt to climate change (Rama Rao et al., 2013). In Karnataka, most northern districts are projected to have more droughts in Kharif season, while eastern districts will experience more droughts in Rabi season (BCCI-K, 2011).

A changing climatic environment also changes the conditions (e.g. higher temperatures, more unpredictable rainfall) that allows for pathogen concentration and disease severity (Gautam et al., 2013). With regards to late blight, climate change will likely increase or reduce the favorable period for the disease to emerge. For instance, in Punjab, the number of favorable days are expected to increase, while in West Bengal the number of favorable days are expected to decrease (Luck et al., 2012, Dua, 2017).

### 4.4 Adoption of resistant and tolerant varieties

**Fig. 1:**
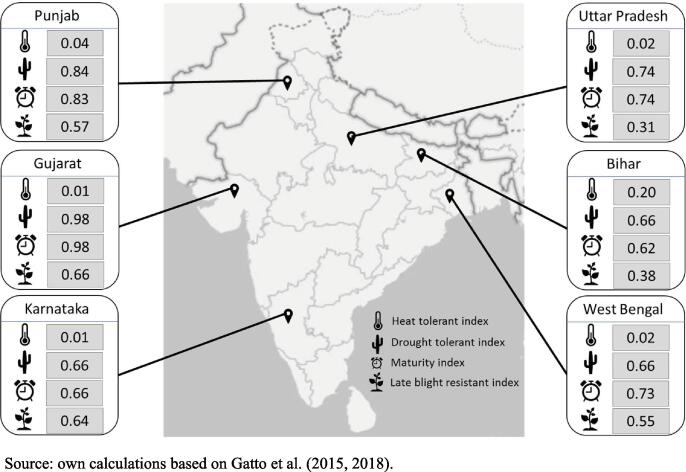
Map of India and Varietal Resilience Indicators for studied states. *Source:* Own calculations based on Gatto et al. (2015), Gatto et al. (2018).

Applying the developed Varietal Resilience Indicator reveals that maturity is one of the most important traits found in adopted varieties in our study region. The Maturity Index (MI) is the lowest in Bihar (0.62) and the highest in Gujarat (0.98). This means that, at the state level, the potato area is dominated by early-maturing rather than late-maturing varieties (Fig. 1). In Gujarat, which had the highest MI, nearly all varieties are early-maturing, with Kufri Pukhraj and Kufri Khyati being the most adopted varieties. Punjab looks similar where more than 80% of the area is planted to early-maturing varieties, more precisely, Kufri Pukhraj is planted in two thirds of the area. Bihar, where 30% of the area is planted to native late-maturing varieties, has the lowest MI score (0.62). Note that this score is relative to other MI scores; in absolute terms, this is still an acceptable score. Finally, in the case of Uttar Pradesh, West Bengal, and Karnataka, most of the area planted to potatoes are medium-maturing varieties. Among the most popular ones are Kufri Jyoti and Kufri Bahar, but also early-maturing varieties, like Kufri Pukhraj.

Drought tolerance is found to be another important trait in the adopted varieties. The Drought Tolerant Index (DTI) appears to be the lowest in Karnataka and West Bengal (0.66) and the highest in Gujarat (0.98). The most adopted variety, Kufri Pukhraj, is early-maturing and highly tolerant to drought, and consequently, states with high adoption of this variety as well as its “improved version”, the Kufri Khyati, have the highest DTI values, such as Gujarat, Punjab and Uttar Pradesh. Similar to the Maturity Index (MI), DTI values are relative, and all states present good DTI scores. Another variety with high drought tolerance is Lady Rosetta, which has been adopted widely in Uttar Pradesh.

The most important varieties planted in India excel on maturity and drought tolerance but do not show high resistance or tolerance levels in other traits. This is the case for the late blight resistance trait, where the top varieties cultivated are either medium resistant or sensitive to late blight. The Late Blight Resistance Index (LBI) is the lowest in Uttar Pradesh (0.31) and Bihar (0.38); and the highest in Gujarat (0.66) closely followed by Karnataka (0.64), making late blight resistance the third most important trait in adopted varieties. The generally low LBI scores are a result of the importance of major varieties, such as Kufri Phukraj and Kufri Jyoti, which only have a medium resistance against late blight. Newer more resistant varieties, such as Kufri Himalini or Kufri Megha, are available but only adopted in some pockets. This in turn may partially be explained by a mismatch between seed requirement and actual supply.

Finally, heat tolerance is by far the least important trait in adopted varieties, as just a few varieties present in India have a high tolerance to heat. The Heat Tolerant Index (HTI) is lowest (equal or less than 0.02) in four states, Gujarat, Karnataka, Uttar Pradesh, and West Bengal; and the highest in Bihar (0.20). Bihar has 31,000 ha (10% of total area) growing Kufri Sindhuri, which helps the state overcome heat stress.

The following potato resilience analysis was conducted after overlaying climate change projections with the Varietal Resilience Indicator.

Punjab has, in relation to other states, the most balanced resilience. High drought tolerance, late blight resistance, and early maturity are found in adopted varieties. Despite the HTI being very low, the state is predicted to become warmer, which will favor potato production. Heat tolerance is thus of less importance. Similarly, due to expected increases in (extreme) rainfall, droughts are unlikely to become an issue for potato production. Given the already high DTI for Punjab (0.84), attention can be redirected to other more important traits in the Punjab context. Late blight will remain an issue given hotter and wetter weather predictions and a current LBI of 0.57.

Gujarat has overall high scores for MI, DTI, and LBI of 0.98, 0.98, and 0.66, respectively. Although climate change predictions likely affect potato production in this region negatively, adopted varieties provide a high degree of resilience. The HTI of Gujarat is 0.01, indicating that heat tolerance will need further attention in the future to prepare this region for predicted warmer weather.

West Bengal also has a relatively balanced varietal resilience. Early maturity is the most important trait here given a MI of 0.73, followed by drought tolerance. As discussed, West Bengal likely benefits from more monsoon rainfall but also appears to have a high DTI of 0.66 which reduced the need for high drought tolerant varieties. In contrast, heat tolerance is only marginally important currently (HTI of 0.02) but warmer weather will likely negatively affect potato production which presents the need to breed and promote varieties with high heat tolerance levels. The LBI score is 0.55, indicating that the late blight pressure will reduce in the future and the need for resistant varieties will reduce.

Karnataka has very similar varietal index scores as West Bengal, but climate change effects the two regions differently. The major difference is that rainfall will likely be reduced, thus prolonging drought periods and therefore negatively affect potato production. However, high drought tolerant varieties are currently adopted as the DTI of 0.66 shows. Late blight resistance is important for Karnataka because most of the potato is grown in the rainy season with high late blight incidence between July and September where no other State can produce varieties suitable for processing (Marwaha et al., 2010). Currently, high late blight resistant varieties are adopted, as shown by an LBI of 0.64 and priorities may shift. For instance, to heat tolerance, which has only received marginal attention as the HTI of 0.01 reveals.

Uttar Pradesh is doing well regarding drought tolerance (DTI of 0.74) and early maturity (MI of 0.74). However, drought is predicted to only become an issue in eastern parts of the State; for the western parts, varietal promotion could prioritize other traits. More specifically, heat tolerance (HTI of 0.02) and late blight (LBI of 0.31) need more attention.

Bihar has, in comparison to other states, the highest heat tolerance (HTI of 0.20). However, in absolute terms high tolerance deserves future attention given the likely negative impact of warmer (extreme) temperatures on potato production. In contrast, predictions are mixed for rainfall but given the relatively high DTI of 0.66, Bihar is well adapted in this respect. More concerning is the low late blight resistance (LBI of 0.38) indicating that late blight pressure will likely increase.

## 5 Conclusions

Breeding and adoption of improved high resistant varieties are an important strategy to adapt to the negative effects of climate change. In this study, we created Varietal Resilience Indicators to analyze adoption rates of improved varieties for four important traits (i.e. heat tolerance, drought tolerance, early maturity, and late blight resistance) and discuss our findings in the light of current climate change projections for our study region. We use the example of the Indian potato sector, focusing on six states, Bihar, Gujarat, Karnataka, Punjab, Uttar Pradesh, and West Bengal.

Even though, different climate change models have shown contradictory patterns in some states like Punjab, and some authors such as Mohanty et al. (2015) predict increase of rainfall extreme events in Gujarat, most climate change literature predict that climate change will negatively affect potato production in most states, in the absence of adaptation strategies. However, in some states the effects of climate change on potato production are positive. For instance, Punjab may benefit from decreasing temperatures and more rainfall. Understanding the impact of climate change, as both a positive and negative scenario, is an important observation which has implications for breeding and varietal promotion activities. For example, in areas positively affected by climate change not all traits are required allowing for prioritization of varietal dissemination.

In using the Varietal Resilience Indicator, we found that in all states high resistant and tolerant varieties are adopted providing some degree of varietal resilience. Early maturity was the most important trait observed in the stock of adopted varieties, followed by drought tolerance, late blight resistance, and heat tolerance. At the state level, we found Gujarat to have the highest share of high drought tolerant, high late blight resistant, and early-maturing varieties. Punjab was found to have similarly high resilience levels. Striking was that heat tolerance has not been widely adopted. This may be a result of heat stress not being an issue currently, however, it will be in the future in all states except Punjab. In addition, new populations of late blight have emerged threatening the (future) potato production in many states (Chowdappa et al., 2015). However, farmers in different states are not investing in new materials with resistance to this pathogen. Special attention needs to be paid to investing in breeding for high heat tolerance and late blight resistant varieties (Marwaha et al., 2010, Rana et al., 2013a) and the subsequent effective dissemination of those varieties to the states, where these improved varieties are needed most.

Improved varieties with resistances and tolerances to abiotic and biotic stresses, however, only present one adaptation strategy among others. Other farming practices, such as drip irrigation or partial root-zone drying allow for higher water-use efficiency (Yactayo et al., 2013, Qin et al., 2018); integrated pest management allows for reducing pesticide applications in dealing with, for instance, late blight (Forbes and Landeo, 2006). A better understanding of what combination of adaptation strategies work under which conditions, would be an interesting avenue of future research. This is especially important because ‘allrounder’ varieties (i.e. varieties with multiple high resistance/tolerance levels) do not exist.

Finally, we found that keeping productivity at current rates will pose a tremendous challenge for policy-makers, breeders, extension agents, and farmers alike and will require continuous investment into genetic improvement and similar efforts in the promotion and dissemination of released varieties. Even if India becomes highly resilient to climate change, producing food at current rates will not suffice with the growing population. Strategies to produce more food will require more attention in the future which will depend upon using resources more efficiently and reducing negative environmental and social implications. Sustainable intensification of rice-agri systems with potato may be one avenue to achieve this (Garnett et al., 2013), but more research is required to better understand the context and conditions conducive for its success.

## Figures and Tables

**Table:** 

	Minimum temperature (°C)		Maximum temperature (°C)		Rainfall (mm)		Potato Yield (t/ha)	
	Current	Change (%)	Current	Change (%)	Current	Change (%)	Current	Change (%)
Bihar	10.5^2^, 20.0^3^	+32.0%^1^, No change^4^	25.3^1^, 31.0^2^	+8.7%^1^, Increasing trend3	1225 ^6^, 1116^5^	−55.2%^6^, +12%^7^, Increasing trend3	25.8^1^, 40.8^8^	−18%^1^, −11.5%^9^
Gujarat	22.0^10^, 21.0^2^	+5.5%^9^, Increasing trend^3^	33.4^9^, 33.8^2^	+2.1%^9^, Increasing trend3	873^4^, 700^9^	+44.6%^5^, Increasing trend3	37.5^7^	−12.7%^7^, −55.1%^8^
Karnataka	19.0^2^	Increasing trend^3^	29.5^2^	No change3	852^4^	Northern: +77.6%^5^ Southern: +9.8%^5^, Decreasing trend3	6.1^11^	−45.7%^8^
Punjab	16.0^2^	Decreasing trend^3^	30.0^2^	Decreasing trend3	747^4^	+52.9%^5^, Decreasing trend3	51.2^7^	−5.3%^7^, +3.7%^8^, −6.5%^12^
Uttar Pradesh	18.8^2^	No change^3^	32.3^2^	No change3	877^4^, 946^13^	Western: +1.5%^5^ Eastern: −4.8%^5^, Decreasing trend3	45.3^7^	−13.4%^7^, +9.1%^8^
West Bengal	20.1^14^, 22.5^2^	+6.5%^12^, Increasing trend^3^	28.912, 31.0^2^	+4.5%12, No change^3^	2203^12^, 1750^15^	+95.2%^5^, +13.7%12, Decreasing trend12	39.2^7^, 17.412	−12.0%^7^, −16.1%^8^, −25.3%12

## Appendix 1 Current values and effect of climate change on minimum and maximum temperature, rainfall, and productivity in Bihar, Gujarat, Karnataka, Punjab, Uttar Pradesh and West Bengal.

^1^Current average minimum and maximum temperature in Bihar is based on potato cropping season in four districts between 2008 and 2010 (Haris et al, 2015).

^2^https://www.currentresults.com/Weather/India/average-annual-temperatures.php for temperature (accessed on November 06th, 2018).

3Rathore et al. (2013) give annual temperature trends on °C/year for the period 1951–2010, but no current values.

4https://www.currentresults.com/Weather/India/average-yearly-precipitation.php for precipitation (accessed on November 06th, 2018).

5Guhathakurta and Rajeevan (2008) only shows trends of rainfall variability but not current values.

6Tesfaye et al. (2017).

7Dua (2017), yield effect of climate change for Kufri Pukhraj variety for year 2055, Karnataka was not part of the study.

8Singh and Lal (2009)/Ayyogari et al. (2014) only show trends in yield but not absolute values.

9Rathore and Jasrai (2013) shows the increment of temperature from 1957 to 2007 in Gujarat.

10Rana et al. (2013b).

^11^Dua et al. (2013) shows a negative effect on productivity in Punjab when including the effect of CO2 concentration.

^12^Government of Uttar Pradesh (2014).

^13^Luck et al. (2012) gives results of temperature, rainfall and yield for potato production using the PRECIS model for West Bengal.

^14^Government of West Bengal (2011).

## Footnotes

1 After 2015 five additional improved varieties have been released (Luthra et al., 2017).
